# PSA testing for prostate cancer: an online survey of the views and reported practice of General Practitioners in the UK

**DOI:** 10.1186/1471-2296-6-24

**Published:** 2005-06-09

**Authors:** Jo Brett, Eila Watson, Paul Hewitson, Colleen Bukach, Adrian Edwards, Glyn Elwyn, Joan Austoker

**Affiliations:** 1 Cancer Research UK Primary Care Education Research Group, Department of Primary Health Care, Old Road Campus, University of Oxford, Headington, Oxford, OX3 7LF, UK; 2 Centre for Health Sciences Research, Cardiff University, 56 Park Place, Cardiff, CF10 3AT, UK

## Abstract

**Background:**

The role of Prostate Specific Antigen (PSA) testing in the early detection of prostate cancer is controversial. Current UK policy stipulates that any man who wishes to have a PSA test should have access to the test, provided he has been given full information about the benefits and limitations of testing. This study aimed to determine UK GPs' current reported practice regarding PSA testing, and their views towards informed decision-making and PSA testing.

**Method:**

Online questionnaire survey, with a sample of 421 GPs randomly selected from a database of GPs across the UK.

**Results:**

95% (400/421) of GPs responded. 76% of GPs reported having performed a PSA test for an asymptomatic man at least once in the previous three months, with 13% reported having tested more than five men in this period. A majority of GPs reported they would do a PSA test for men presenting with a family history and requesting a test, for asymptomatic men requesting a test and also for men presenting with lower urinary tract symptoms. Reported testing rates were highest for men with a family history. Amongst men with lower urinary tract symptoms and men with no symptoms, reported testing rates were significantly higher for older than younger men.

The majority of GPs expressed support for the current policy (67%), and favoured both the general practitioner and the man being involved in the decision making process (83%). 90% of GPs indicated that they would discuss the benefits and limitation of testing with the man, with most (61%) preferring to ask the man to make a further appointment if he decides to be tested.

**Conclusion:**

This study indicates that PSA testing in asymptomatic men is a regular occurrence in the UK, and that there is general support from GPs for the current policy of making PSA tests available to 'informed' men who are concerned about prostate cancer. While most GPs indicated they would discuss the benefits and limitations prior to PSA testing, and most GPs favoured a shared approach to decision making, it is not known to what extent men are actually being informed. Research is needed to evaluate the most effective approach to assisting men in making an informed decision about whether or not to have a PSA test.

## Background

The increasing incidence of and mortality from prostate cancer has led to widespread calls for prostate cancer screening. However, the subject is controversial [1-3]. PSA testing has high false positive rates and there is the potential for over-diagnosis of slow growing prostate cancers that may never present as a problem. Furthermore there is no strong evidence that treatments for localised cancer are effective, and no good evidence at present that screening would result in a reduction of mortality. Screening could therefore result in more harm than good [1].

Randomised trials are currently underway in Europe and the US to assess the impact of screening [4]. Definitive information from these trials will not be available until later this decade. Recent falls in mortality in the US [5], where there is widespread PSA testing, are often cited as being indicative of successful screening. However, interpretation of this data is complex, with factors such as lead-time bias (where PSA testing has prolonged the length of time a patient is identified as having the disease without prolonging their life) and length bias (where PSA testing has resulted in increased diagnosis of slow-growing or non-progressing tumours with a good prognosis) contributing to this outcome [6].

In the UK, the National Screening Committee has recommended that a prostate cancer screening programme should not be introduced at this time [7]. However, in response to growing public concern about prostate cancer, in 2001 the Department of Health introduced a Prostate Cancer Risk Management Programme (PCRMP) [8], which provides men with access to the PSA test, provided they have been given full information regarding its' possible benefits and limitations.

While rates of PSA testing in England and Wales have been assessed previously [9], there is very little evidence to date regarding general practitioners' (GPs) current practice in testing for prostate cancer. Furthermore, while surveys conducted in Australia, NZ and USA have reported GPs' knowledge and opinions of PSA testing for prostate cancer [10-14], little is known about GPs' current views towards PSA testing in the UK. One survey conducted in 1998 with GPs in the North Staffordshire district of England found that 81% (134/167) GPs agreed there should not be a population screening programme at that time [15]. However, 57% (93/163) felt that if patients are educated about the benefits and limitations then they should be allowed to make their own decision about being tested. Only 9% (15/168) of GPs in this survey reported testing asymptomatic men, although nearly two-thirds of respondents reported that they or their staff were asked about prostate cancer screening by their patients.

The main aims of this study were to determine GPs' current reported practice regarding PSA testing, and to assess their views regarding current policy on informed decision-making and PSA testing.

## Methods

A questionnaire was developed and piloted with a sample of 30 GPs from the Oxfordshire GP research consortium and GP contacts in Wales and Hertfordshire in September 2003. The survey was hosted by MEDIX, a web-based internet service provider for doctors . MEDIX has a user group of 12,000 doctors, with 4,700 GPs registered from a wide demographic base and with a wide distribution in terms of decade qualified. Male GPs are over-represented in the database (79%). No remuneration or incentives were offered for participating in this survey (although GPs registered with MEDIX are offered incentives for participating in certain types of online research). A random sample of 421 GPs from the MEDIX database were e-mailed an invitation to take part in the survey. The e-mail contained a link to the questionnaire which addressed GPs' reported practice and views using a combination of direct questions and 5 consultation vignettes. The first two vignettes described men aged 55 and 70 years respectively who presented with mild lower urinary tract symptoms (LUTS). There was no mention in the vignette that the men had raised the issue of prostate cancer or PSA testing. The third man presented with concerns about his family history of prostate cancer (his father died aged 75 of prostate cancer and his brother had been diagnosed last week) and asked to be tested for prostate cancer. The fourth and fifth men were asymptomatic (aged 55 and 70 years respectively), but had both recently lost a friend to prostate cancer, and asked to be tested for prostate cancer. The survey was posted on 24/11/03. All responses were received within 30 hours.

## Results

### The participants

The response rate was 95% (400/421). Of these, 94% (375) were partners in a GP practice, 4% (16) were GP assistants, and 2% (9) were GP registrars. The majority were full time (86%, 343), and male (82%, 327). There was a wide coverage by region across the UK (for example 15% (59) from the South-East, 9% (36) from the South-West, 10% (39) from the North West, 12% (49) from Scotland and 5% (21) from Wales). There was also a broad range from decade qualified, with 3% (14) qualified in the 1960s, 31% (125) qualified in the 1970s, 43% (173 qualified in the 1980s, 21% (85) qualified in the 1990s, and 1% (3) qualified in the 2000s. When compared with national statistics for GPs [16], female GPs, as expected, were significantly under-represented in this sample (18% vs 36%), as were non-partner GPs (6% vs 15%).

### General Practitioners' reported practice

Table 1 below reports the tests which GPs reported they would elect to conduct for each of the vignettes. GPs were significantly more likely to report they would do a PSA test for a man who presents with concerns about his family history and requests a test, than for men who present with either mild lower urinary tract symptoms (LUTS) (p < 0.0001) or asymptomatic men who request a test (p < 0.0001). GPs were also significantly more likely to report they would do a PSA test for a 70 year old than for a 55 year old man either presenting with LUTS, or requesting a test in the absence of symptoms. GPs also frequently reported they would do a digital rectal examination (DRE) either if the man requested a test because of his family history, or presented with mild lower urinary tract symptoms. Less than half of the GPs reported they would do a DRE if the man was asymptomatic, but were significantly more likely to do one for a 70 year old than a 55 year old asymptomatic man in this situation.

**Table 1:** Reported use of tests by general practitioners (N = 400)

**Vignette**	**Mild lower urinary tract symptoms (55 years)**	**Mild lower urinary tract symptoms (70 years)**	**Family history, asymptomatic, requests test (55 yrs)**	**Asymptomatic, requests test (55 yrs)**	**Asymptomatic, requests test (70 yrs)**
	% *(95% CI) *n	% *(95% CI) *n	% *(95% CI) *n	% *(95% CI) *n	% *(95% CI) *n
**Prostate Specific Antigen (PSA)**	60% *(55,64) *239	78% *(73,82) *311	91% *(87,93) *363	60% *(55,65) *239	71% *(66,75) *282
**Digital rectal examination (DRE)**	69% *(64,73) *274	78% *(73,81) *310	67% *(62,72) *268	37% *(32,42) *147	47% *(42,52) *189
**Trans-rectal ultrasound**	2% *(1,4) *9	7% *(4,10) *27	10% *(7,13) *41	1% *(0,2) *2	2% *(0,4) *7
**No tests**	6% *(4,9) *25	3% *(2,5) *13	3% *(1,5) *11	22% *(18,26) *88	14% *(11,18) *58
Other*	47% *(42,52) *188	41% *(36,46) *165	14% *(11,18) *56	17% *(14,21) *69	13% *(10,16) *51

*Other mainly includes mid-stream urine, other urinary checks and discussion / counselling.

When asked approximately how many asymptomatic men they had discussed PSA testing for prostate cancer with in the past 3 months, 12% (48) reported none, 65% (259) reported discussions with between 1 and 5 men, 17% (68) reported discussions with between 6 and 10 men, and 6% (25) reported discussions with more than 10 men.

Twenty-four percent (94) of GPs said they had not conducted any PSA tests for asymptomatic men in the past three months, 63% (252) reported they had tested between 1 and 5 asymptomatic men, 9% (37) reported between 6–10 men, and 4% (17) reported more than 10 men.

Regarding the role of practice nurses in the PSA testing process, 76% (304) said nurses were not involved, 16% (63) said the nurse conducts PSA testing following counselling from the GP, and 4% (16) reported that nurses conduct both counselling for the test and taking the sample.

### General practitioners' views towards PSA testing

67% GPs responded that they supported the current policy. When specifically asked to indicate their preference for a national PSA testing policy, six percent (22) GPs believed PSA testing should not be available to asymptomatic men and 33% (132) GPs would prefer selective screening of high risk men. 8% (31) GPs said they would like to see the introduction of a population screening programme. The remainder supported patient or GP initiated screening, or a combination of both. No associations were found between years practising as a GP or gender of GP and the views expressed above.

### Informed decision-making

When asked how GPs would prefer men to make their decision regarding whether or not to have the PSA test, 49% (196) said the man should make the final decision after seriously considering his GP's opinion, 34% (135) said the GP and the man should share the responsibility, 13% (51) said the man should make the final decision completely by themselves, 4% (17) said the GP should make the final decision, after seriously considering the man's opinion, and 1% (2) said the GP should make the final decision.

GPs also indicated how they would prefer to conduct a consultation regarding PSA testing – 55% (219) of GPs reported they would prefer to discuss the benefits and limitations of PSA, provide the man with written information, and request that the man makes another appointment if they decide to be tested; 35% (140) of GPs reported they would provide the counselling and then perform the PSA test within the same consultation if the man still wishes to be tested; 5% (20) said they would conduct the test without counselling, but explain why they are conducting the test, and 3% (13) would reassure the man that he did not need a PSA test.

## Discussion

This study of UK GPs indicates that PSA testing of asymptomatic men is a common occurrence, and that consultations which involve a discussion of PSA testing often result in the test being performed. Most GPs in the study reported they would perform a PSA test for men concerned about their family history of prostate cancer. Most reported they would also test men who present with mild LUTS, or who request a 'screening' test in the absence of any symptoms – particularly older men. An Australian study has also suggested that age and LUTS may provide 'cues' to action for PSA testing [16]. The distinction between a 'screening' and 'diagnostic' PSA test tends to become blurred as men get older and the presence of some degree of urinary symptoms becomes increasingly common. Even if urinary symptoms are present there is disagreement amongst experts as to whether the PSA test should be routine or whether men should 'opt in' to having the test [17]. Urinary symptoms are most often caused by benign prostatic hypertrophy (BPH), and there is a very weak evidence base for the primary care diagnosis of prostate cancer in men with LUTS [18]. Early, localised prostate cancer usually does not produce symptoms and by the time prostate cancer is causing urinary symptoms it is likely to have reached an advanced stage, where treatment options are reduced. Some men will have a co-existing early prostate cancer and BPH, and when a man seeks advice about LUTS this can set in train investigations which diagnose a coincidental prostate cancer. Coping with a positive PSA test performed with no prior warning could be distressing for some men, and research is needed to explore the level of counselling occurring in men with LUTS prior to PSA testing in primary care.

Although the current policy recommendation in the UK is that a DRE is not warranted in asymptomatic men [19], almost half of the GPs in the survey indicated they would perform a DRE for an asymptomatic 70 year old man concerned about prostate cancer, and over a third for a younger man. Previous studies have also reported endorsement of use of both the PSA and DRE in asymptomatic men [20,21], and it has been suggested that this may be because the DRE is seen as a routine component of a physical examination for men [12]. The American Cancer Society has also recommended the use of both [22], although the US Preventive Services Task Force concludes that the evidence is insufficient to recommend for or against routine screening for prostate cancer using PSA or DRE [23].

In line with the reported practice of GPs, the survey found that whilst GPs do not support the introduction of a national PSA screening programme, there is support for the current policy of providing men with access to the test, provided they have been given prior information on the benefits and limitations. Most respondents in this survey favoured both the GP and the man sharing in the decision-making process and the largest proportion indicated they would prefer to counsel men first and then take the blood sample during a separate consultation. Facilitation of informed decision-making is a key element of policy, but there is currently little evidence about how best to organise services to achieve an informed decision. One study reported that conducting prenatal screening in the same appointment, as opposed to requiring a return visit, may lead to higher levels of informed choice [24]. Another study assessing cystic fibrosis screening found patient choice was based on more knowledge when testing was part of a separate visit [25]. Separating information provision from PSA testing would require more consultation time, and would be less convenient for a man who knows he wants to be tested. Additionally, there may be a perception that because testing was not offered at the time the GP does not really support testing. Conversely, with same-day testing the patient may be more influenced by the GP's opinions and may not have had sufficient time to fully understand and consider the benefits and limitations.

Research is needed to assess the most effective way of achieving and measuring informed decision making for PSA testing. In addition, attempts to study the informed decision-making process need to take into account the different influencing factors. As well as knowledge and the way testing is organised, personal attitudes and values, the attitudes of health professionals, and social and media influences can all affect the decision.

This study has some limitations. The sample was drawn from a database of GPs registered with one particular internet service provider and whilst previous studies have demonstrated the acceptability of the internet as a resource for medical research in primary care [26], there are also recognised drawbacks [27]. The main limitation of the study is the potential for selection bias. As we expected from the profile of GPs registered with MEDIX, in comparison with national GP statistics [28] female doctors are under-represented in our study. We do not, however, believe this to be a major issue for this particular topic as anecdotal evidence from many GPs (both male and female) would indicate that the majority of consultations which include a discussion of prostate issues occur with male GPs. In support of this, some recent pilot work we have conducted found that 90% of GP responders to a survey sent out following a request for a PSA test were male. The sample is, however, also likely to have over-represented internet literate GPs who may not be representative of the wider GP community. A further important limitation of the study is the use of consultation vignettes to seek GP's reported behaviour. The vignettes can only provide minimal information about the patient / consultation, sometimes making expected behaviour difficult to predict. We also acknowledge the potential difference between reported and actual behaviour with respondents more likely to report what they perceive to be 'appropriate' behaviour than what they may actually do in practice. It is important that future studies in this area address the actual behaviour of GPs.

Overall PSA testing rates in the UK are rising [29]. Although the exact proportion of asymptomatic men being tested, and the extent to which this is patient driven, is unknown, a recent study reports that the rate of asymptomatic testing in general practice is at least 1.6% per annum and higher if private testing is included [9]. With the increasing media attention being given to prostate issues it is expected that these rates will continue to rise and it is therefore important there is adequate consideration of the implications for both primary and secondary care. The Prostate Cancer Risk Management Programme has circulated an information pack on this topic to GPs , but it is likely that a multi-faceted approach is required for the successful dissemination of this information to primary care [30].

## Conclusion

This study indicates that PSA testing in asymptomatic men is a regular occurrence in the UK, and that there is general support from GPs for the current policy of making PSA tests available to 'informed' men who are concerned about prostate cancer. While most GPs in the study indicated they would discuss the benefits and limitations prior to PSA testing, and most GPs favoured a shared approach to decision making, it is not known to what extent men are actually being informed. Research is required to determine the most effective approach to assisting men in making an informed decision about whether or not to have a PSA test.

## Competing interests

The author(s) declare that they have no competing interests

## Authors' contributions

JB participated in the design and conduct of the study, and co-wrote the manuscript; EW participated in the study design and co-wrote the manuscript; PH participated in the study design and writing the manuscript; CB participated in the study design and writing the manuscript; AE participated in the study design and writing the manuscript; GE participated in writing the manuscript; JA participated in the study design and writing the manuscript. All authors read and approved the final manuscript.

## Pre-publication history

The pre-publication history for this paper can be accessed here:


